# Manipulating the confinement of electromagnetic field in size-specific gold nanoparticles dimers and trimers†

**DOI:** 10.1039/c9ra07346a

**Published:** 2019-12-19

**Authors:** Sudip Kumar Pal, Hirak Chatterjee, Sujit Kumar Ghosh

**Affiliations:** Department of Chemistry, Assam University Silchar-788011 Assam India sujit.kumar.ghosh@aus.ac.in +91-3842-270848

## Abstract

The intriguing light–matter interactions can be governed by controlling the particle size and shape, electromagnetic interactions and dielectric properties and local environment of the metal nanostructures. Amongst the different approaches that have been engendered to manipulate light at the nanoscale, the self-assembly of metallic nanostructures with controllable interparticle distances and angular orientations, which strongly impact their optical attributes, is one of the viable avenues to exploit their utility in a diverse range of niche applications. The simplest geometrical architectures that enable such modulations are dimers with changeable interparticle distances and trimers with an additional degree of angular orientation to correlate the plasmonic observables with the observed spectral characteristics. Wet chemical approaches have been adopted in this study for the synthesis of size-selective gold nanoparticles, and appropriate organic linkers have judiciously been employed to induce plasmonic interactions amongst the gold nanoparticles in close proximity to each other. The combination of experimental observations and electromagnetic simulations adopted to probe the plasmonic interactions revealed that the electrodynamic coupling effect was very sensitive to particle size, interparticle distances and angular orientations in these simple nanoassemblies. The capability to precisely manipulate the electric field at the junctions between these plasmon-coupled nanoparticles could pave the way for the application of these nanoassemblies in surface-enhanced spectroscopies and sensing applications.

## Abstract 2

Confinement of the electromagnetic field in gold nanoparticle dimers and trimers with variations in the interparticle distances and angles has been calculated.

## Graphical abstract

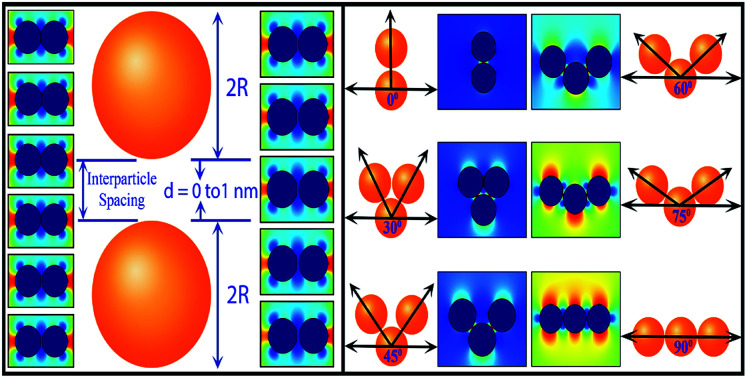

## 1 Introduction

The collective properties of an ensemble of plasmonic nanostructures are significantly altered than the algebraic sum of all their individual properties, and this leads to the emergence of communal properties in the system.^1^ Amongst the different approaches that have been engendered to venture the plasmonic attributes, the assembly of nanostructures offers the avenue for varying degrees of plasmon coupling, eventually concentrating the light into the nanometer-scale volume between neighbouring nanostructures.^2^ The ability of noble metal nanoparticles in manipulating the position, orientation, and connectivity of colloidal building blocks is intrinsic to manipulate light at the nanoscale, as has been obvious in a diverse range of niche practical applications ranging from biosensing to miniature optoelectronic devices.^3,4^ Upon interaction with incident electromagnetic radiation, the collective oscillations of the conductive electrons at the subwavelength dimensions lead to localised surface plasmon resonances (LSPRs), and the resonance condition critically depends on the particle size, shape, interparticle coupling, dielectric properties and local environment of the nanostructures.^2–4^ When the metallic particles in the dispersion are sufficiently isolated, the shape, size and dielectric properties of the surrounding medium dramatically influence the position, bandwidth, and intensity of the confined field, as well as the resonance energy of the nanostructures. However, when two or more nanostructures are situated in close proximity (*d* ≤ 5*R*, where *R* is the radius of the spherical nanoparticles, and *d* is interparticle separation distance) to each other, the electrodynamic coupling of the plasmons gives rise to a new set of plasmonic modes, as well as coherence and decoherence of the electromagnetic field.^5^ While tuning the band maximum to the desired wavelength is necessary for the amplification of the extinction cross-sections and optical fields in the preferred regions of the electromagnetic spectrum, the collective optical properties of the aggregates of metallic nanoparticles can be modulated by controlling the geometric arrangements, such as the interparticle distances and relative orientations, of the assembly.^2–5^ Thus, exploring the fundamental nature of plasmon coupling by spatially controlling the interparticle distance and/or angle is of immense significance from both the theoretical and experimental perspectives.

Because of the difficulty in governing the kinetics and the lack reproducibility thermodynamically stable aggregates, plasmonic dimers and trimers can be selected as the simplest model nanoantenna to elucidate the electromagnetic coupling effect as a function of interparticle distance and angle the building units. Various strategies, including electron beam lithography,^6^ high-purity separation,^7^ DNA base pairing,^8^ molecular bridging,^9^ photoirradiation,^10^ electrostatic interactions,^11^ and mechanical strain,^12^ have been employed in the literature for the spatial manipulation of gold nanoparticle dimers and trimers with ideal (spherical) geometry. When two or more nanoparticles are placed sufficiently close to each other to form an assembly, the Coulomb interaction becomes strong enough to alter the distribution of surface charges between the particles. The plasmonic aspects of the gold nanoparticle dimers^6,13–15^ and trimers^16–18^ have been unraveled from both theoretical and experimental perspectives. The observed spectral irregularity of non-ideal (faceted) building blocks^19–22^ is because the relative orientations of the corners, edges, and faces create various types of fixtures at the junctions between the particles. Several physical attributes, for example, emission polarisation,^23^ polarisation of the incident light,^24^ non-local effects,^25^ Landau damping,^26^ and Fano interference,^27^ have also been observed as a consequence of the plasmon coupling of the gold nanoparticle dimers and trimers. The plasmonic interactions between the nanostructures that strongly localises the charges at the junctions of the particles imbues dramatic impact in materials science, such as non-linear optical properties,^28^ surface-enhanced Raman spectroscopy,^29^ surface-enhanced fluorescence,^30^ two photon excitation photoluminescence,^31^ plasmonic rulers,^32^ optical trapping,^33^ plasmonic sensing,^34^ nanoscale photonic switches,^35^ chiroplasmonic activity,^36^ DNA cleavage dynamics,^37^ and photothermal therapy of cancerous cells.^38^ Different types of theoretical formalisms, such as generalised multiparticle Mie theory,^39^ phenomenological surface collision damping theory,^40^ hydrodynamic model including diffusion,^41^ self-consistent theory,^42^ jellium model,^43^ plasmon hybridisation theory,^44^ local analogue model,^45^ dressed-states picture,^46^ and group symmetry approach^47^ have been adopted for an accurate description of these assemblies and to investigate their local and non-local attributes. Numerous computational techniques, such as discrete dipole approximation (DDA),^48^ finite element method (FEM),^49^ finite-difference time-domain (FDTD) method,^50^ boundary element method (BEM),^51^ and multipole expansion methods (MEM),^52^ have been explored for studying the electrodynamics of such assembled nanostructures. Despite the significant advances in theoretical modeling, simulation techniques and experimental strategies, the understanding of plasmon coupling for size-selective particles in close proximity to each other is of great significance.

In this article, we aim to elucidate the possibility of confinement of electromagnetic field when the particle size is larger (in the regime of 8–25 nm) compared to the interparticle spacing, particularly at the subnanometer-scale distances (≤1 nm). Size-selective gold nanoparticles in the specified dimensions were synthesised by the Frens' citrate reduction procedure, and aggregation amongst the gold nanoparticles was induced by suitable organic linkers, *viz.* 1,8-diaminooctane and phloroglucinol, to selectively form dimers and trimers, respectively. Dimers and trimers have been chosen as the simplest model systems so as to systematically investigate the effect of interparticle distances and angular orientations, respectively, on the plasmonic coupling of these assembled nanostructures. The experimental observations have been complemented by employing T-matrix simulations and the finite element method to correlate the plasmonic perspectives and for the field enhancement of these assemblies. Therefore, the present article investigates the plasmonic attributes by varying the interparticle distances and angular orientations of the size-selective gold nanoparticle dimers and trimers when the particle size is very large compared to the interparticle distance. The concept that the trimer can be considered as a special case dimer from the viewpoint of angular orientations and simulation based on the superposition T-matrix method are firsts in a study on these kinds of systems. The physical signature of electromagnetic coupling between the neighbouring particles forms the basis for deciphering the morphological and orientational changes, suggesting future means to manipulate light at the nanoscale.

## 2 Experimental methods

### 2.1 Reagents and instruments

All the reagents used were of analytical grade. Choloroauric acid (HAuCl_4_·3H_2_O), 1,8-diaminooctane, phloroglucinol (1,3,5-trihydroxybenzene) and trisodium citrate were purchased from Sigma-Aldrich and used as received. Spectroscopy-grade solvents, *viz.* ethanol and tetrahydrofuran (THF) were obtained from Qualigens' Fine Chemicals, India and used without further purification. Milli Q deionised water was used throughout the course of the investigation.

The absorption spectra were recorded in a PerkinElmer Lambda 750 UV-vis-NIR digital spectrophotometer with 1 cm well-stoppered quartz cuvettes for samples. Transmission electron micrographs (TEM) of the metal colloids were captured with a Hitachi H-9000NAR transmission electron microscope operating at 200 kV. The samples were prepared by mounting a drop of each of the respective dispersions on carbon-coated copper grids and subsequent drying in the air. High-resolution transmission electron micrographs (HRTEM) were obtained using the same instrument. Dynamic light scattering (DLS) studies were performed using a NanoZS (Malvern) instrument after filtering the colloidal dispersions using a Millipore syringe filter (0.2 mm pore size). The zeta potentials of the gold particles before and after the addition of organic ligands were measured by using a Nano-ZS (Malvern) test measurement system. Dark-field imaging was carried out using an Olympus GX 51F inverted optical microscope by illuminating the samples with a halogen light source (U-LH100-3). The images of single nanostructures were placed at the entrance of an imaging spectrometer (SpectraPro 150, Acton Research), and the spectra were detected with a thermoelectrically cooled CCD camera (Prosilica GE680C). An Ocean Optics Flame-T-UV-VIS-ES spectrometer connected with the dark field microscope was used to measure the single particle scattering spectra. Calculations were performed by the Finite element method using the COMSOL Multiphysics software package. Superposition T-matrix method was adopted to compute the extinction profiles of the monomeric gold nanoparticles and their assemblies using the freely available JaSTA® software package.^53^ The electric field of the incident light was polarised both along and perpendicular to the interparticle axis. The optical constant of bulk gold provided by Johnson and Christy^54^ was used in all calculations. Meshgrid structures (assuming a mesh size of 0.16 nm^2^) were obtained using FreeCAD®, Meshlab® and ImageJ software packages. The refractive index of the medium was set to 1.333 to mimic the aqueous environment.

### 2.2 Preparation of gold nanoparticles monomers, dimers and trimers

Monodispersed gold colloids over a wide size range were synthesised by the Frens' citrate reduction procedure,^55^ as described in ESI 1.† The particles formed by this method were spherical or nearly spherical with the average diameters of 8 ± 0.7, 10 ± 0.9, 16 ± 1.3, 20 ± 1.8 and 25 ± 2.3 nm for sets A–E, respectively. The details of the synthetic conditions and characteristic parameters of the size-specific gold nanoparticles are enunciated in Table 1.

**Table 1:** Summary of the synthetic conditions and characteristics of the gold colloids^a^

Set	Amount of HAuCl_4_ solution (10 mM, mL)	Amount of trisodium citrate solution (1%, mL)	Color	*λ*_max_ (nm)	Particle size^b^ (nm)
A	1.25	2.0	Dark red	518	8 ± 0.7
B	1.25	1.6	Red	519	10 ± 0.9
C	1.25	1.0	Red	522	16 ± 1.3
D	1.25	0.875	Red	526	20 ± 1.8
E	1.25	0.750	Red	528	25 ± 2.3

a The total volume of the solution was maintained at 50 mL for all sets of particles.

b The particle size is represented as the mean diameter ± the standard deviation of the particle size distribution.

Purification of the gold nanoparticles was carried out by the repeated centrifugation and redispersion of a 2.0 mL aqueous dispersion of citrate-stabilised gold nanoparticles (0.25 mM) thrice and final dispersion in 1.0 mL of ethanol by sonication. Then, 1.0 mL of 1,8-diaminooctane (0.1 mM) was diluted with a mixture of 0.4 mL ethanol and 0.6 mL THF, and to this solution, 1.0 mL of the ethanolic dispersion of gold nanoparticles was added drop-wise. The resultant dispersion was shaken for 3 min (to avoid the formation of larger aggregates), and the colour changed from red to yellowish brown, indicating the formation of dimers of the particles. A similar procedure was followed for all the five different sized gold particles.

Similarly, 1.0 mL of phloroglucinol (0.1 mM) was diluted with a mixture of 0.4 mL ethanol and 0.6 mL THF, and to this solution, 1.0 mL ethanolic dispersion of gold nanoparticles was added drop-wise. The resultant dispersion was stirred for 3 min (to avoid the formation of larger aggregates), and the colour changed from red to dark brown, indicating the formation of trimers of the gold nanoparticles. A similar procedure was followed for all the five different sized gold particles.

### 2.3 T-matrix calculations

The superposition T-matrix method solves Maxwell's equations for the multiple spherical particles by matching the boundary conditions.^56^ In this method, the scattered electromagnetic field arising from the ensemble of N spherical particles is denoted by the superposition of the electromagnetic fields that are scattered from each of the spheres present in the ensemble or the aggregate. The field arising from each individual sphere is represented by an outgoing vector spherical wave harmonic expansion from the center of the sphere. The T-matrix method utilises the Lorentz–Mie technique to solve the scattering problem for each sphere and subsequently, considers the superposition of the fields arising from the ensemble of spheres. The superposition T-matrix method is a very fast and accurate numerical technique. Therefore, for an aggregate of spheres, this method is one of the best techniques compared to the discrete dipole approximation (DDA), which assumes that the particles consist of point dipoles and is much time-consuming.

## 3 Results and discussion

In this experiment, gold nanoparticles were prepared by the Frens' citrate reduction procedure, and due to the adsorption of the negatively-charged citrate anions onto the gold surface, the particles carried a residual surface charge that stabilised them.^55^ Upon the addition of either 1,8-diaminooctane or phloroglucinol into the colloidal dispersion of gold, the colour rapidly changed from red to yellowish brown or dark brown, respectively, indicating aggregation between the particles. According to the classical Derjaguin–Landau–Verwey–Overbeek (DLVO) theory,^57^ the pairwise interaction forces arise due to the interplay of the attractive van der Waals forces and the repulsive Coulomb forces screened by the Debye–Hückel ion clouds and under the condition that the attractive forces dominate over the repulsive forces (*F*_attr_ ≫ *F*_rep_), leading to aggregation amongst the monomeric building units. The lack of sufficient surface charge renders the particles aggregate, and therefore, selective aggregation could be induced upon the addition of appropriate organic linkers to replace the labile citrate ions from the particle surface *via* ‘place exchange reactions’.^2^ For example, the monomeric gold nanoparticles (set D) showed an average zeta potential of −35.3 mV, which increased to +7.6 and +3.9 mV for the dimers and trimers, respectively. Before the addition of organic ligands, the gold particles are stable in the colloidal dispersion since the energy barrier is high enough to prevent aggregation. Upon the addition of specific organic molecules, the energy barrier is lowered significantly, leading to destabilisation and subsequent aggregation amongst the particles. While two or three nanoparticles come in close proximity to each other, the Coulomb interactions between the particles are strong enough to alter the surface charge, and the judicious manipulation of charge distribution causes the selective conjugation of monomeric building units to dimers and trimers.

**Fig. 1:**
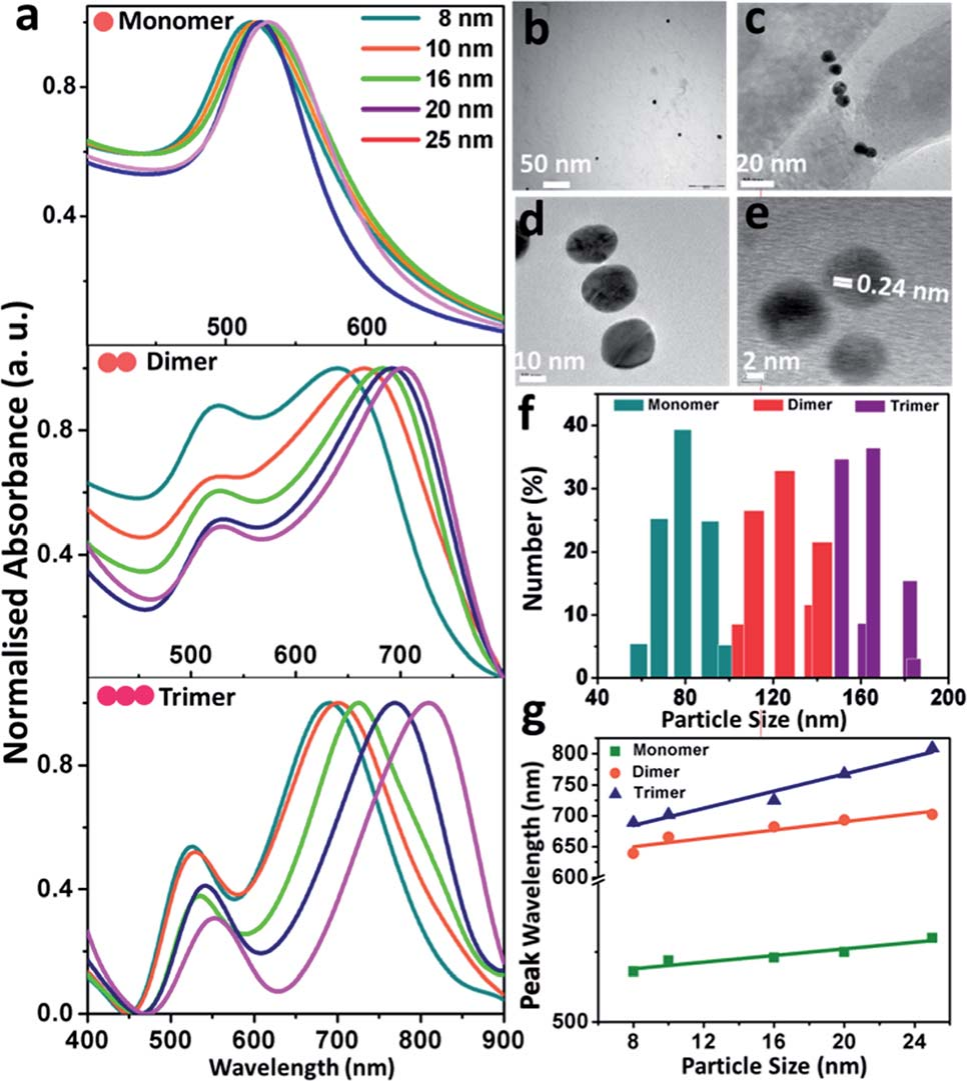
Absorption spectral and morphological characteristics of the size-selective gold nanoparticles and their assemblies: (a) normalised localised surface plasmon band of the ensemble-averaged gold nanoparticle monomers, dimers and trimers; (b, c and d) representative transmission electron micrographs of the gold nanoparticle (set D) monomers, dimers and trimers, respectively; (e) high-resolution transmission electron micrograph of a representative gold nanoparticle (set B) trimer; (f) dynamic light scattering profile showing the relative size of the gold nanoparticle (set B) monomers, dimers and trimers; (g) profile showing the comparison of shift in the peak wavelength as a function of particle size corresponding to the monomers, dimers and trimers, respectively. The shift of the longitudinal band has been taken into consideration for the dimers and trimers.

The absorption spectral and morphological features of the size-selective gold nanoparticles and their assemblies are shown in Fig. 1. Fig. 1a exhibits the ensemble-averaged absorption spectra of the size-selective gold nanoparticle monomers, dimers and trimers. The absorption spectra of monomeric gold nanoparticles of variable sizes (Fig. 1a, top) corresponding to the localised surface plasmon resonance bands appear with maxima at 518, 519, 522, 526 and 528 nm, respectively. Naked eye observation shows that the colour of the dispersions changed from dark red to red with an increase in the size of the particles. It was observed that monomeric dispersion of the gold particles exhibited only a single peak, and as the particle size increased, the wavelength of maximum absorption gradually shifted towards the higher wavelength regime. Nevertheless, upon the addition of the organic ligand, the plasmon band showed the appearance of two distinct absorption maxima: one at the wavelength corresponding to the maximum of the monomers and the other broadened band at a red-shifted wavelength, indicating electrodynamic coupling between the neighbouring particles forming the assemblies.^2–4^ The salient feature of physical significance was that the variation in particle size led to the fine tuning of the plasmon coupling amongst the nanoparticles. As the particle size increased, the first peak showed a continuous decrease in intensity but remained at nearly the same wavelength, while the second peak shifted to higher wavelengths. The first band was assigned to the transverse plasmon coupling mode, while the second band could be attributed to the longitudinal plasmon coupling along the interparticle axis, as explained by the concept of plasmon hybridisation.^44^ Careful observation showed that while the transverse band shifted only from 519 to 530 nm, the longitudinal band red-shifted from 639 to 702 nm as the size of the individual particles increased from 8 to 25 nm in the dimers (Fig. 1a, middle). Weak coupling between the plasmon dipoles of each nanoparticle perpendicular to the interparticle axis could have led to nearly the same energy as the plasmon maximum of the individual gold nanoparticles, while the longitudinal band could be attributed to plasmon coupling along the interparticle axis and was sensitive to the size of the particles.^13^ The splitting of the surface plasmon energy could be attributed to the formation of strong electromagnetic interactions between the nanoparticle pairs, which lead to the polarisation-dependent splitting of plasmon resonance, and the interparticle dipole–dipole interactions were apparently exhibited by the gradual red-shift of the band with an increase in the size of the particles. The absorption spectra of the trimers (Fig. 1a, bottom) showed that the transversal band appeared nearly in the same region, while the longitudinal band shifted more towards the higher wavelengths than that of the dimers with increasing size of the monomeric building units. Fig. 1b–d display the representative transmission electron micrographs of the gold nanoparticle (set B) monomers, dimers and trimers, respectively; the images of dimers and trimers visually exhibit the appearance of dielectric separation between the pairs of particles. Fig. 1e shows the representative high-resolution transmission electron micrograph of the trimeric gold nanoparticles (set D) with the lattice fringes of 0.24 nm corresponding to the (111) plane of fcc structured gold, which indicated that the morphology of the individual gold particles was retained upon the formation of the assemblies. Statistical analyses of the size distribution histograms corresponding to the monomers, dimers and trimers were further obtained from dynamic light scattering experiments (Fig. 1f). They revealed that the average hydrodynamic diameter of the particles gradually increased with subsequent aggregation into dimers and trimers compared to the monomeric building units. The profile showing the plot of the maximum absorption wavelengths as a function of particle diameter (Fig. 1g) revealed that the slope increased, indicating the formation of highly efficient coupled structures with an increase in the extent of aggregation between the particles.

**Fig. 2:**
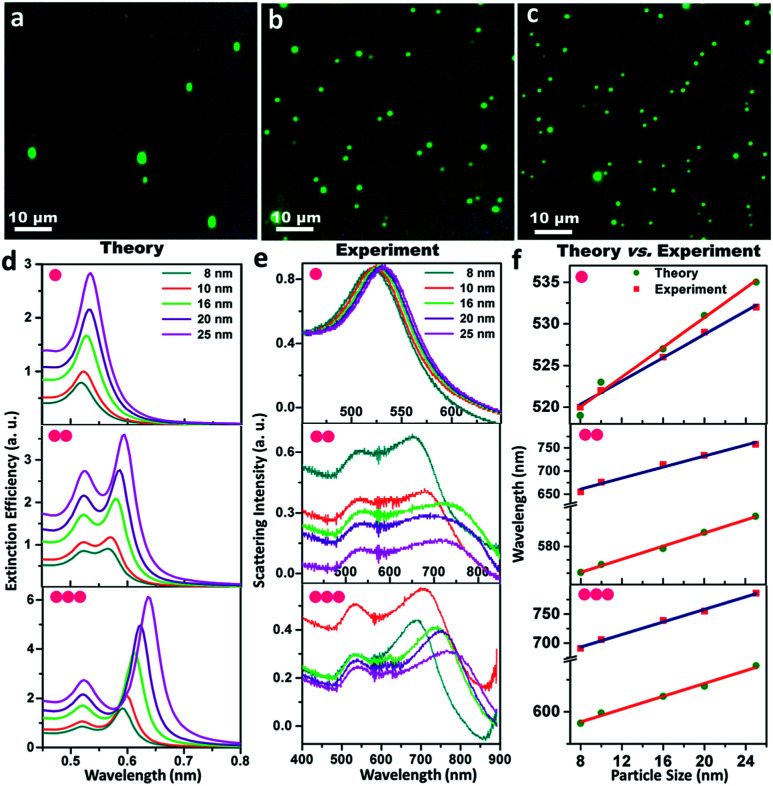
Single particle scattering spectroscopy and electrodynamic simulations of the size-selective gold nanoparticles and their assemblies: (a, b and c) dark-field microscopic images of the gold nanoparticle (set D) monomers, dimers and trimers, respectively; (d) theoretical scattering spectra based on T-matrix simulations; (e) experimental scattering spectra and (f) the comparison of the wavelength of absorption maximum as a function of particle size for the gold nanoparticle monomers, dimers and trimers, respectively. The peak maxima of the longitudinal bands have been taken into consideration for dimers and trimers.

The monomeric gold nanoparticles and their assemblies were visualised using dark field microscopy and consequently, characterised using single particle scattering spectroscopy from both theoretical and experimental viewpoints, as presented in Fig. 2. Fig. 2a–c display the representative dark field microscopic images (using a green filter) of the gold nanoparticle (set D) monomers, dimers and trimers, respectively; the micrographs of the dimers and trimers exhibit reasonable precision for the interparticle separation within the assemblies. Electrodynamic simulations based on the T-matrix method were performed to calculate the extinction spectra of the monomers, dimers and trimers, as shown in Fig. 2d. The array of monomeric building units in the dimers and trimers was assumed to be linear; as the interparticle separation was varied in the range of 0 to 1.0 nm (discussed in detail in the following section), the average was taken as 0.5 nm for the simulations. In addition to the fundamental absorption peaks in the range of ∼520 nm, new absorption bands appeared at higher wavelengths with maxima in the regime of ∼580 and ∼620 nm for the dimers and trimers, respectively. The new peaks observed in the T-matrix simulations at the higher wavelength regime could be attributed to the typical longitudinal dipolar plasmon coupling of the multipeak plasmonic spectra of the assembled metal nanoparticles. Moreover, a continual increase in intensity and narrowing of the extinction peak were also observed at the higher wavelength regime. It was observed that when the excitation polarisation was along (0°) and perpendicular (90°) to the axis of the assemblies, the polarised spectra revealed red- and blue-shift of the maxima from plasmonic oscillations through the long and short axes, respectively. Then, the optical responses of the size-specific individual monomers, dimers and trimers were monitored by single-particle dark field scattering spectroscopy, as exhibited in Fig. 2e. While one plasmon resonance peak was observed in the range of ∼540 nm for the gold nanoparticle monomers, additional resonance peaks at longer wavelengths manifested in the regime of 652–753 and 691–786 nm corresponding to dimers and trimers, respectively. The difference between the five different sized gold nanoparticles originated from the excitation of the hybridised surface plasmon modes that are parallel to the long axis of the dimers and trimers arranged in a linear fashion with constant interparticle separation and manifested as the characteristic red-shift from the monomer plasmon resonance. Even though the emergence of scattering spectral features due to the difference in interparticle separation was obvious, the general difference between the single particle spectroscopic images of the monomers, dimers and trimers was apparent. Comparison between the theoretical and experimental scattering spectra showing the peak maximum (*λ*_max_) as a function of the particle diameter for the size-selective gold nanoparticle monomers, dimers and trimers, respectively, are shown in Fig. 2f. The peak maxima corresponding to the typical single resonance of the monomers and the longitudinal dipolar plasmon bands of the dimers and trimers were taken into consideration. It was noted the trend in the appearance of the maximum wavelength was similar to that predicted by the T-matrix simulations. However, the observed anomaly between the simulated and observed spectral evolutions could be attributed to the differences in the parametric assignments for the interparticle distances and size distributions in the simulations as opposed to the experimental observations corresponding to the particular sets of gold nanoparticles. When the neighbouring particles were coupled, the resonant interaction led to the evolution of two orthogonal modes: a blue-shifted transverse mode and a red-shifted longitudinal mode. However, it was also taken into consideration that in strongly interacting small metallic particulates, it is plausible that the higher order modes of the particles can mix with each other. Such an admixture of higher order modes in the assemblies may lead to plasmon excitation by light in the spectral region of the higher energy antisymmetric dipolar modes. To clarify this ambiguity, deconvolution across the scattering spectra of the gold nanoparticle (set A) dimers and trimers was carried out by global fitting, as described in ESI 2.† On assuming the individual spectral features to be Lorentzian, the interpolation lines corresponded to the well-resolved transverse and longitudinal modes of the dimers and trimers and ruled out plausible contributions by the higher order modes.

**Fig. 3:**
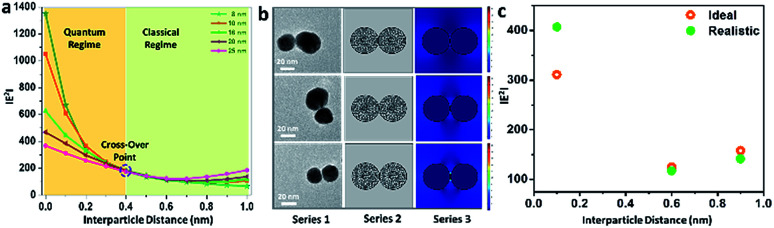
(a) Profile showing the variation of |*E*^2^| as a function of the interparticle distance of the size-selective gold nanoparticle dimers; (b) representative real-time transmission electron micrographs of the gold nanoparticle (set E) dimers, the corresponding mesh-grid structures and the associated electromagnetic field enhancement represented as series 1, 2 and 3, respectively; (c) profile showing the comparison between the |*E*^2^| values obtained under ideal and realistic conditions for the manifestation of results under analogous experimental conditions.

Further, electromagnetic field simulations of a series of precisely-aligned configurations of the gold nanoparticle dimers were performed by varying the interparticle spacing in the sub-nanometer regime (≤1 nm separation) in chronological order through the finite element method, as displayed in ESI 3.† The hybridised plasmonic modes emerging from electromagnetic coupling were viewed similar to the formation of lower energy “bonding” and higher energy “antibonding” modes in analogy to the notion of molecular orbitals.^44^ The electric field distribution patterns for bonding and antibonding modes with increase in interparticle distance show that the electric field in the interparticle space is localised along the line through the contact edge of the monomeric building units, and the higher intensity plasmon modes appeared near the vertices. As the interparticle separation decreased, the intensity of the electric field became increasingly intense for all particles of different sizes. The results revealed two opposite trends, as evident from the calculated |*E*^2^| values, for the bonding and antibonding modes. In the first case, when the dimer axis was aligned with the electric field direction, it showed a consecutive increase in field strength with increasing particle size, and when in perpendicular alignment, it decreased linearly. In order to validate the simulation results, the calculated values of the enhancement of electromagnetic fields of the gold nanoparticle dimers were compared with similar parametric assignments observed in the transmission electron micrographs, as presented in Fig. 3. Fig. 3a enunciates the variation in the calculated |*E*^2^| values for the size-selective gold nanoparticle dimers by the finite element method as a function of interparticle separation. The salient feature of physical significance was that the electromagnetic field increased continually as the interparticle distance, *d*, decreased and abruptly increased beyond a critical interparticle distance (*d*_c_) until the two nanostructures nearly touched each other. Interestingly, the field enhancement was greater for smaller particles beyond the optimum interparticle distance (∼0.4 nm), and cross-over interaction was observed at the critical point. These observations revealed that the electromagnetic coupling strength correlated with the enhancement of the electric field between the neighbouring particles. The calibration curves identified two distinct plasmon coupling regimes: classical (*d* > *d*_c_) and quantum (*d* < *d*_c_), suggesting that the quantum effects are prevalent over the classical electromagnetic capacitive charges beyond the critical distance between the two adjacent metal nanoparticles.^14,58,59^ Therefore, it was apparent that beyond the critical interparticle distance, the electric field polarisation increases drastically for a particular size regime. This observation emphasised that smaller nanoparticles are viable for light absorption, and above a particular size, the particles are more appropriate for light scattering experiments. When the interparticle distance was decreased in a spatially controlled manner beyond the threshold point of separation, the electromagnetic field enhancement could be attributed to the generation of ‘hot spots’ between the particles approaching the immediate vicinity, effectively minimising to a small mode volume between the building units of the dimers. To examine the validity of the model in calculating the electromagnetic field confinement, the interparticle spacings of the gold nanoparticle (set E) dimers measured from the transmission electron micrographs were compared to the results from the simulation performed under analogous experimental conditions (Fig. 3b). The enlarged versions of the transmission electron micrographs of the gold nanoparticles dimers are displayed in ESI 4.† A comparison between the simulations under ideal and realistic parametric assignments (Fig. 3c) validated that the model agreed well with the calculated electromagnetic field enhancement for the dimers.

Different aspects have been investigated by eminent research groups with regard to the variations in interparticle separation by employing the classical electrodynamic theory, as well as the quantum theory of plasmonics. Experiments conducted by Schultz and co-workers^5^ have shown that for interparticle distances of the order of the nanoparticle size, the plasmon spectral peak, when excited with light polarised along the dimer axis (longitudinal polarisation), red-shifted exponentially with a decrease in the interparticle distance. Aussenegg and co-authors^43^ have found that for the light polarised perpendicular to the dimer axis (transverse polarisation), the plasmon resonance blue-shifted. However, for large interparticle separations, the results could be well-described by dipolar interactions. Schatz and colleagues^60^ have shown that higher order terms are important for the quantitative description of plasmon spectra of dimers with smaller interparticle distances. Khlebtsov group^52^ systematically compared the plasmon spectra of dimers with various interparticle distances obtained using dipolar approximation or full electrodynamic calculations, including all multipole orders, and demonstrated the significance of the multipole orders for an accurate description of the plasmon fields. Nerkararyan and co-workers^61^ solved the wave equation for two conductive spheres in the immediate vicinity, predicting huge enhancement of the electromagnetic energy at the exact point of contact. Nijhuis and colleagues^62^ directly observed the charge transfer plasmon mode in the electron energy loss spectra of a silver nanoparticle dimer with the nanogap narrowed by electron beams or molecular linkers, revealing quantum plasmon interactions. Therefore, the extent of the electromagnetic interaction is a sensitive function of the interparticle distance; as the interparticle distance decreases, the interaction between the nanoparticles strengthens, and the electric field increasingly localises at the junctions between the nanoparticles.

**Fig. 4:**
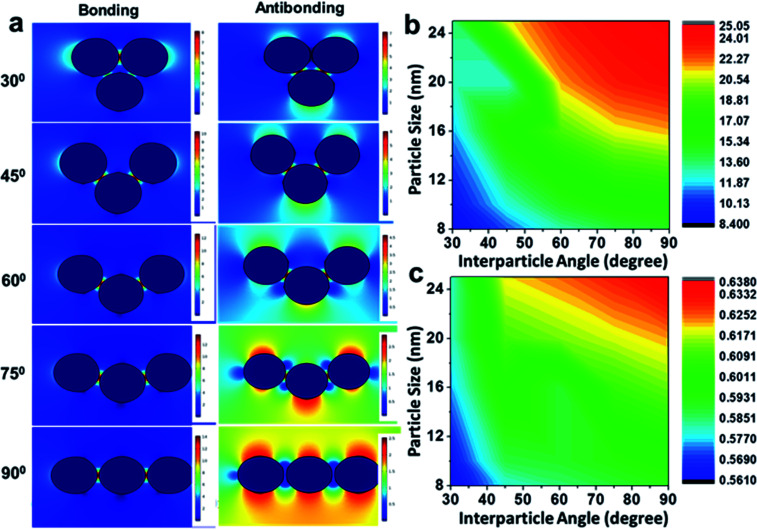
(a) Electric field distribution pattern in the bonding and antibonding modes with an increase in the interparticle angle of the gold nanoparticle (set B) trimers calculated by the finite element method; and (b and c) profiles showing the total displacement of the electric field with the variation of particle size, as well as interparticle angle, of the gold nanoparticle trimers in the bonding and antibonding modes, respectively.

Although an ideal dimer consisting of two spherical building units at a constant separation distance is just a simple geometrical configuration, a trimer represents a more complex plasmonic system with the possibility of variations in the interparticle angles as additional degrees of freedom. Therefore, minute alterations in the angular orientations (0° < *θ* < 90°) alters the direction of light propagation in trimeric metal nanostructures. Fig. 4 represents the simulation of the electric fields defined by the finite element method with a periodic assignment of the spatial coordinates of the terminal spheres with respect to the initial linear axis, keeping the central sphere fixed at the origin. Optical simulations considering the electromagnetic coupling in each set of the bent trimers of the homogeneous spheres were performed with varying interparticle angles, *viz.* 30°, 45°, 60°, 75° and 90° angular orientations, as displayed in Fig. 4a. It was observed that as the interparticle angle increased from *θ* = 30° to 90°, the polarisation followed the *a* cos^2^ *θ* function in the azimuthal plane between the assembled nanostructures and reached the maximum at a 90° orientation of the terminal spheres, illustrating the superposition of the modes. Fig. 4b and c enunciate the surface plots corresponding to the directionality of the field polarisation of the bonding and antibonding modes for the size-selective gold nanoparticle trimers. It was observed that |*E*^2^| increased with an increase in both the orientation angles and particle sizes and exhibited the highest enhancement in the electric field at the perpendicular orientation of the trimer. This was because of the fact that as the interparticle angle increases, the two plasmon modes shift towards each other eventually merging into a single, more localised and intense mode that is concentrated in the interparticle space between the vicinal nanostructures.^63^

The series of extinction spectra as a function of an interparticle angle for the different particle sizes simulated using the superposition T-matrix method is presented in ESI 5† assuming an average edge-to-edge separation of 0.5 nm. It could be seen that both the transversal and longitudinal bonding modes were sensitive to the interparticle angles, although the longitudinal bonding mode was the dominant component in the spectra. The scattering simulation also showed an unprecedented blue-shift of the transverse mode for the orientational configuration of trimers at 45°, which corroborates the coexistence of energetically degenerated bonding and antibonding states in their coupled characteristics. It is, thus, evident that slight conformational changes in the gold nanoparticle trimer can lead to miniature the plasmon excitations on the metallic nanostructures.

Moreover, we have performed an explicit analysis of the electric field distribution patterns of the bonding and antibonding modes for the orientational control of the gold nanoparticles trimers. The electric field distribution patterns of the representative bonding modes with gradually increasing interparticle angle calculated by the finite element method are presented in ESI 6,† covering all plausible configurations between an equilateral triangle and a linear chain of the size-specific gold nanoparticle trimers, have been. It could be inferred that the enhancement of electric field is largely dependent upon the angular orientations, which is governed by the morphology of the gap region and decreases with an increase in the size of the particles.

**Fig. 5:**
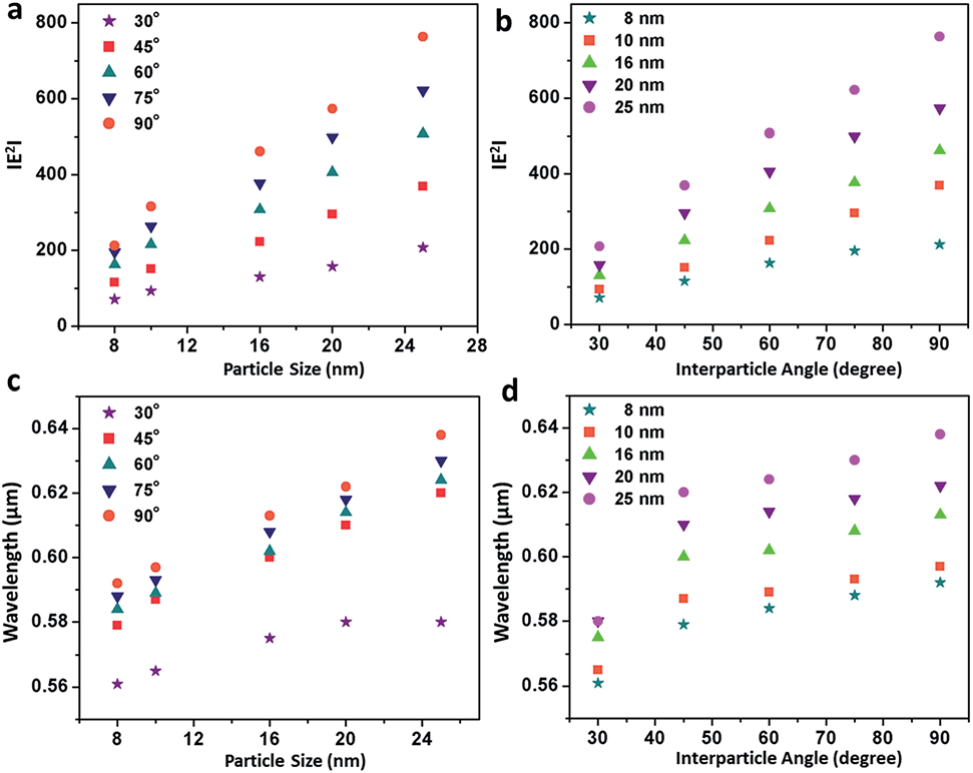
Profiles showing the variation in the electric field and peak wavelength of the size-selective gold nanoparticle trimers: (a and b) linear fits showing the variation in |*E*^2^| as a function of particle size and interparticle angle, respectively, calculated by the finite element method; (c and d) exponential fits of the variation in peak wavelength as a function of particle size and interparticle angle, respectively, computed by T-matrix simulation.

Fig. 5 summarises the profiles showing the variation in the electric field and peak wavelength to illustrate the universal scaling of the plasmon coupling of the size-selective gold nanoparticle trimers. A critical analysis shows that although the electric field distributions can be represented by a good linear fit as a function of both particle size and interparticle angle, the peak wavelength dependence could be best described by a single exponential function in the classical electromagnetic coupling regime. This indicated that the plasmonic excitation energy decreased and the electromagnetic field enhanced with the increase in particle size and interparticle angles. Therefore, manipulation of interparticle angle in gold nanoparticles trimer could be rendered for plausible engineering through light manipulation in these simplest nanoassemblies.

**Fig. 6:**
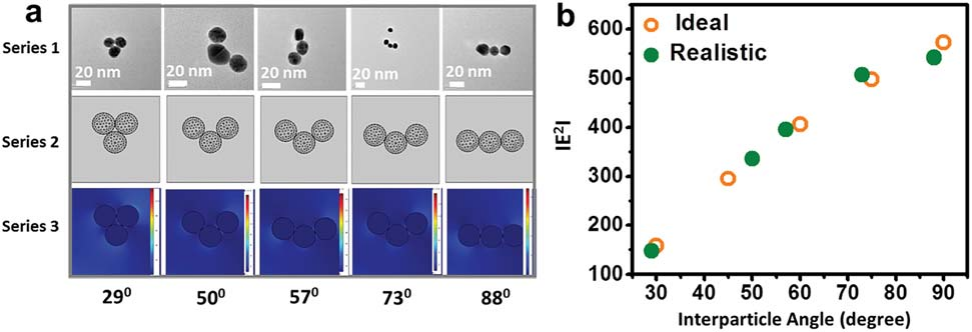
Correlation of the electric field enhancement of gold nanoparticle trimers through parametric assignment: (a) representative real-time transmission electron micrographs of the gold nanoparticle (set D) trimers, the corresponding mesh-grid structures and associated field enhancement represented as series 1, 2 and 3, respectively; and (b) profile showing the comparison between the |*E*^2^| values under ideal and realistic conditions for the manifestation of results under analogous experimental conditions.

Fig. 6 elucidates the correlation of field enhancement with angular assignments adopted from specific transmission electron micrographs of gold nanoparticle (set D) trimers using the finite element method. The morphological description of the nanoassemblies with different interparticle angles was simulated to compare the results from analogous experimental conditions (Fig. 6a). The enlarged view of the transmission electron micrographs of the gold nanoparticles trimers is displayed in ESI 7.† It revealed that the simulation results of the gold nanoparticle trimers with variations in the interparticle angle under ideal and realistic conditions were comparable (Fig. 6b), indicating that the electric field localisation in these simplest nanoassemblies can plausibly be engineered.

**Fig. 7:**
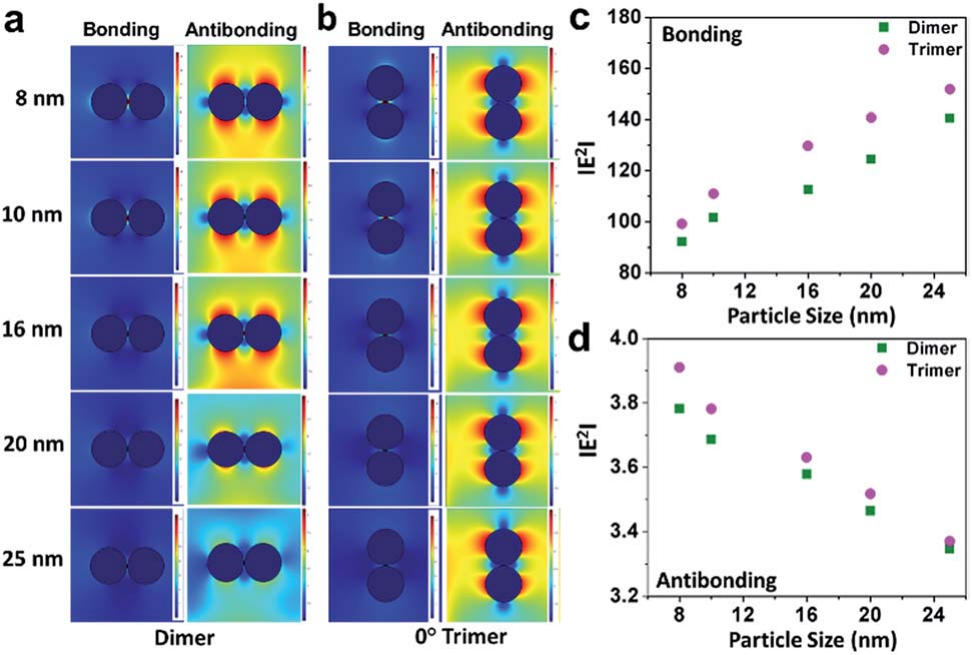
(a and b) Comparison of the electric field distribution patterns in the bonding and antibonding modes of dimers and 90° drifted trimers (where two particles can be assumed to superimpose on each other), respectively, corresponding to the gold nanoparticles of five different sizes calculated by the finite element method; (c and d) profiles showing the linear variation in |*E*^2^| as a function of particle size for the bonding and antibonding modes of the gold nanoparticle dimers and trimers, respectively.

For a general outlook, a comparative study of the electric field distribution was performed considering dimers and a linear chain of trimmers, as shown in Fig. 7. Fig. 7a and b elucidate the difference in the electric field polarisation of the bonding and antibonding modes for the dimers and 90° drifted trimers (where one particle is supposed to eventually merge to the central sphere such that the morphology becomes converted from L- to I-shape), respectively. It was seen that, for the bonding molecular mode (Fig. 7c), the electric polarisation increased with an increase in particle size.

Moreover, the field polarisation by the dimer-like trimer was different from the electromagnetic response of the structures. It is interesting to note that the field polarisation was slightly higher, which shows that electromagnetic coupling is more sensitive at this particular orientation than that of the dimers. On the contrary, for the antibonding mode (Fig. 7d), the slope decreased because of the electric field from another axis perpendicular to the bonding mode axis. This could be ascribed to the additional contribution from the third particle although it is present in a dimer-like geometry. Since a trimer possesses two electromagnetic hotspots through the vicinal interactions with the terminal nanospheres, they produce a higher resultant field than the dimers. While the particles are aligned in the direction of the incident field, the contribution of electromagnetic field results in a greater enhancement of the local field, leading to a sharp linear increase than that of the dimers, whereas the perpendicular arrangement shows a sharper linear decrease of the resultant field due to the reversal in the direction of the local field generated by the trimers compared to that of the dimers.

## 4 Conclusions

In conclusion, we performed a comprehensive experimental and electrodynamic simulation approach to unravel a picturesque description of the electromagnetic coupling effect in gold nanoparticle dimers and trimers. It was observed that the plasmonic attributes, as well as the confinement of electromagnetic fields, were sensitive functions of the distance and relative orientations of the monomeric building units because of the confinement of the interaction space. It was apparent that larger nanoparticles at subnanometer gaps produced the ideal dimeric and trimeric structures, enabling the manipulation of the plasmon line shape over a wider spectral range and producing highly localised and enhanced electric fields in the interparticle space, which suggests a future means for the precise control of light polarisation at the nanoscale. It is interesting to note that the coupling strength in the dimers correlated well with the interparticle separation with cross-over interaction within the electron tunneling range and was dependent on the size regime of the particles. It also revealed that the spectral features were very sensitive to the relative orientations in the trimers, and an abruptness was observed at the 45° angular orientation of the particles. A significant variation in the electric field polarisation is also manifested even when the third particle in the trimer drifted to form the dimer, illustrating the importance of fine tuning the assemblies to achieve desired optical characteristics. A critical correlation of field enhancement was performed through the assignment of parameters observed in the transmission electron micrographs for the manifestation of results under analogous experimental conditions. The combined experimental and theoretical notions elucidate the possibility of manipulating light at the nanoscale with these simplest assemblies, which can be adopted for surface-enhanced spectroscopies and sensing applications in the future.

## Conflicts of interest

There are no conflicts to declare.

## Supplementary Material

RA-009-C9RA07346A-s001
