# Supplementary material for: Manipulating the confinement of electromagnetic field in size-specific gold nanoparticles dimers and trimers
Source: RSC Adv. 2019 Dec 19;9(72):42145–54. doi: 10.1039/c9ra07346a (PMC9076545; doi:10.1039/c9ra07346a)
Supplement: RA-009-C9RA07346A-s001 [file RA-009-C9RA07346A-s001.pdf]

Electronic Supplementary Material (ESI) for RSC Advances.
This journal is © The Royal Society of Chemistry 2019

Electronic Supplementary Information

Manipulating confinement of electromagnetic field in size-specific gold nanoparticles dimers and trimers

Sudip Kumar Pal, Hirak Chatterjee and Sujit Kumar Ghosh*

Department of Chemistry, Assam University, Silchar-788011, India

E-mail: sujit.kumar.ghosh@aus.ac.in; Tel: +91-3842-270848

ESI 1. Synthesis of size-selective gold nanoparticles

Monodispersed gold colloids over a wide size range could be achieved by Frens' citrate reduction method (G. Frens, *Nature*, 1973, 241, 20–22). In this method, it is possible to control the size of the particles by varying [Au (III)]/[citrate] ratio during the reduction step. A standard procedure for the preparation of set E is as follows. A 50 mL aqueous solution of $HAuCl_4$ (0.25 mM) is heated to boiling and 0.75 mL of trisodium citrate (1%) is added. In about 25 s, the boiling solution turns faintly blue (nucleation). After approximately 70 s, the blue color suddenly changes to a brilliant red color, indicating the formation of gold nanoparticles. The particles formed by this method were allowed to incubate for ~24 h before further use. The particles formed by this method are spherical or nearly spherical with average diameter *ca.* 8±0.7, 10±0.9, 16±1.3, 20±1.8 and 25±2.3 nm for sets A–E, respectively.

ESI 2. Deconvolution of the localised surface plasmon band of gold nanoparticles dimers and trimers

The deconvolution by globally fitting across the scattering spectra of the gold nanoparticles (set A) dimers and trimers has been carried out as shown in Fig. S1. The individual spectral features were assumed to be Lorentzian, where the band width (full width at half maximum) and energy could be treated as global variables. The interpolation lines correspond to the well resolved transverse and longitudinal modes of the dimers and trimers and rules out the plausible contributions by the higher order modes.

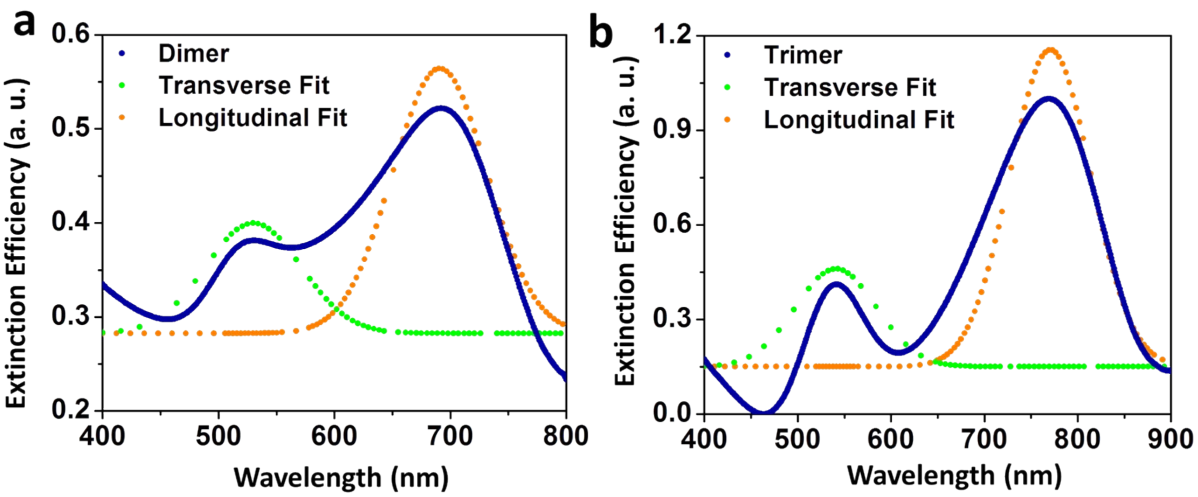

Fig. S1. Deconvolution corresponding to transverse and longitudinal contributions of the localised surface plasmon resonance band of the gold nanoparticles (set A) (a) dimers and (b) trimers.

ESI 3. Electric field distribution patterns for bonding and antibonding orbitals with variation of interparticle separation in gold nanoparticles dimers

The electromagnetic field simulations of a series of precisely aligned configurations of the gold nanoparticles dimers have been investigated with varying interparticle separation in chronological order for size-specific gold nanoparticles through finite element method. Since the building blocks in ideal dimers are spherical, there is just a single geometrical configuration for the two spheres at a constant gap distance. The polarisation of the electric field along the interparticle axis requires less energy for excitation and an attractive interaction results in the stabilisation of the resonance; on the contrary, the polarisation perpendicular to the interparticle axis leads to a higher energy as a result of repulsive interaction between the adjacent particles and therefore, it becomes obvious to elucidate the mode polarisation dependence in an explicit manner. Fig. S2 shows the electric field distribution patterns for bonding and antibonding orbitals with increase in interparticle distance in the subnanometer regime (≤ 1 nm separation)

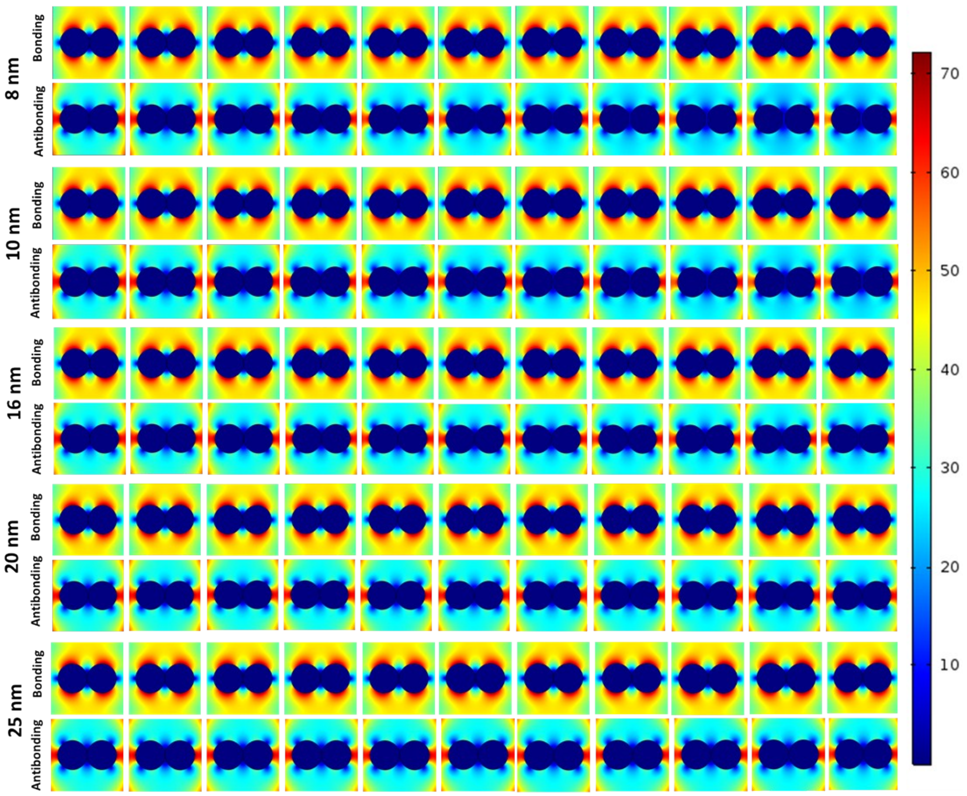

Fig. S2. Electric field distribution patterns for bonding and antibonding orbitals with variation of interparticle separation in the range of 0 to 1.0 nm with a step spacing of 0.1 nm for size-selective gold nanoparticles dimers.

for different sizes of gold nanoparticles. The intensity could be expressed as the modulus square of the corresponding electric field, $|E|^2$, scattered isotropically from the spherical nanoparticles that can be assumed as perfectly Rayleigh scatterers. It is observed that the electric field in the interparticle space is localised along the line through the contact edge of the monomeric building units and the higher intensity plasmon modes appear near the vertices. This is indicative of strong coupling because of the confinement of the electric field within the interparticle spacing between the neighbouring particles. Moreover, the two plasmon modes shift towards each other in accordance with the relative change in vertex positions, eventually, merging into a single, more localized and intense mode that is focused mainly in the overlapping region. It reveals that the control over interparticle separation allows gradual change in the localisation of the electric field and fine tuning of the modes. As the interparticle separation decreases, the intensity of electric field becomes more and more intense for all the different sizes of the particles. The results show two opposite trends as are evident from the calculated $|E^2|$ values, for both bonding and antibonding orbitals. In the first case, when the dimer axis is aligned with the electric field direction, it shows a consecutive increase in field strength with increasing particle size; while, on the perpendicular assignment, it decreases linearly.

ESI 4. Enlarged view of the transmission electron micrographs of the gold nanoparticles dimers

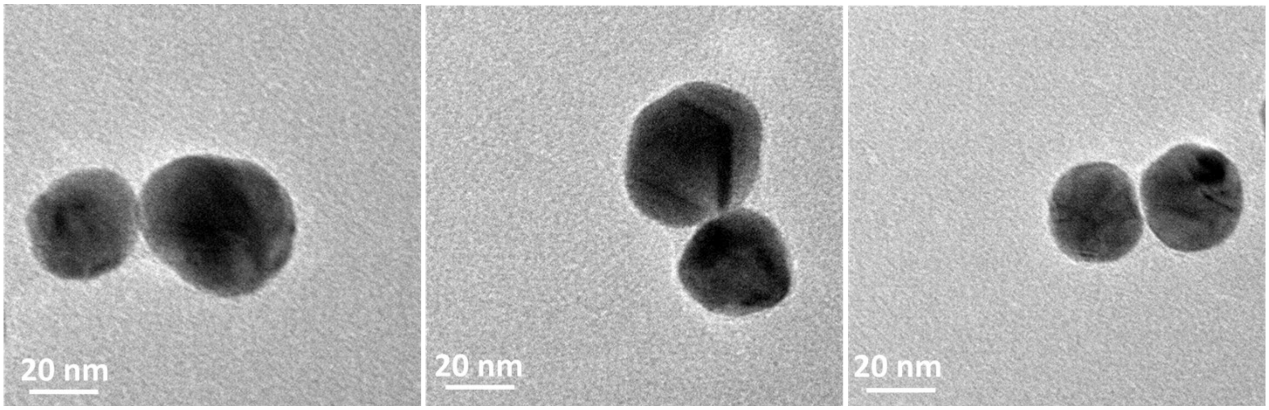

Fig. S3. Enlarged view of the transmission electron micrographs of the gold nanoparticles (set E) dimers

ESI 5. Calculation of the extinction spectra at different interparticle angles corresponding to the size-selective gold nanoparticles trimers

The series of extinction spectra as a function of interparticles angle for different sets of particle sizes 8, 10, 16, 20 and 25 nm for each angular configuration assuming an average of edge-to-edge separation of 0.5 nm simulated through superposition T-matrix method have been exhibited in Fig. S4. The scattering simulation shows an unprecedented blue shift in the transverse mode for the orientational configuration of trimers at 45^0, which corroborates to energetically degenerate and coexisting bonding and antibonding states in their coupled characteristics. It is also obvious that both the transversal and longitudinal bonding modes are sensitive to the interparticle angles, although the longitudinal bonding mode becomes dominant component in the spectra. It is, thus, evident that slight conformational changes in the gold nanoparticles trimer, could led to miniature the plasmon excitations on the metallic nanostructures.

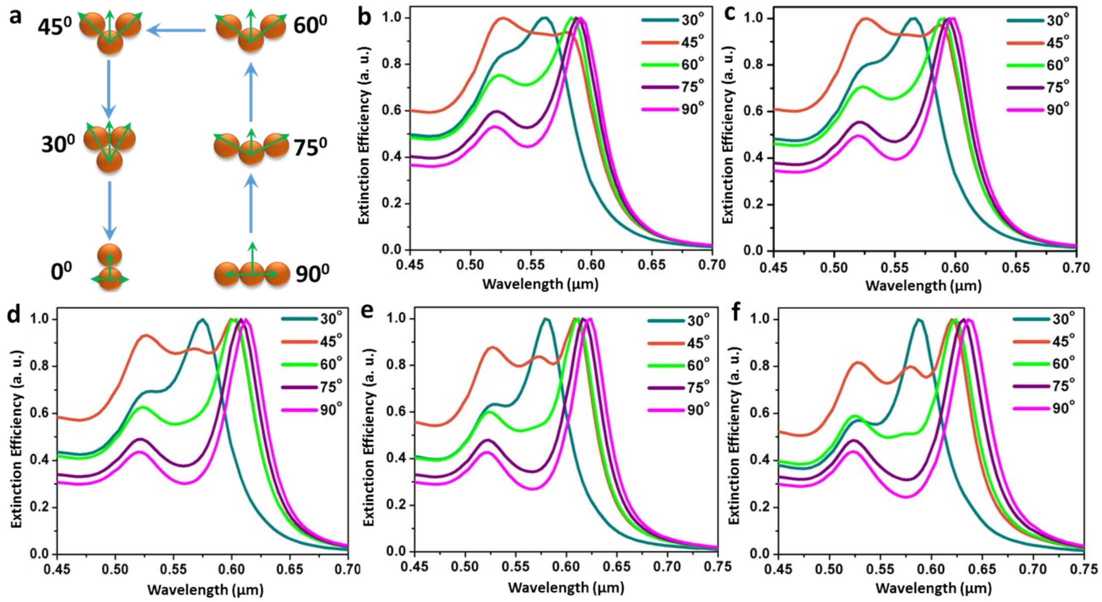

Fig. S4. (a) Schematic presentation showing the gradual changes in the interparticle angle in gold nanoparticles trimes; and (b-f) extinction spectra at different interparticle angles corresponding to the 8, 10, 16, 20 and 25 nm gold nanoparticles, respectively obtained through T-matrix simulation.

ESI 6. Electric field distribution pattern for bonding orbitals with increase in interparticle angle of size-specific gold nanoparticles trimers

We have performed an explicit analysis of the electric field distribution patterns of the bonding and antibonding orbitals for the orientation control of the gold nanoparticles trimers. Fig. S5 shows the electric field distribution pattern for the representative bonding orbitals with gradually increasing interparticle angle, covering all plausible configurations between an equilateral triangle and a trimeric linear chain, of size-specific gold nanoparticles trimers calculated through finite element method. Two important observations contrary to each other could be enunciated: firstly, increase in interparticle angle allows three-dimensional localisation of the electric field due to large accumulation of opposite electric charges in the interparticle region of each individual particle pairs; and secondly, the intensity of the electric field decreases with increase in size of the particles. Therefore, it could be inferred that enhancement of electric field is, largely, dependent upon the interparticle angle governed by the morphology of the gap region and while decreases with increase in size of the particles.

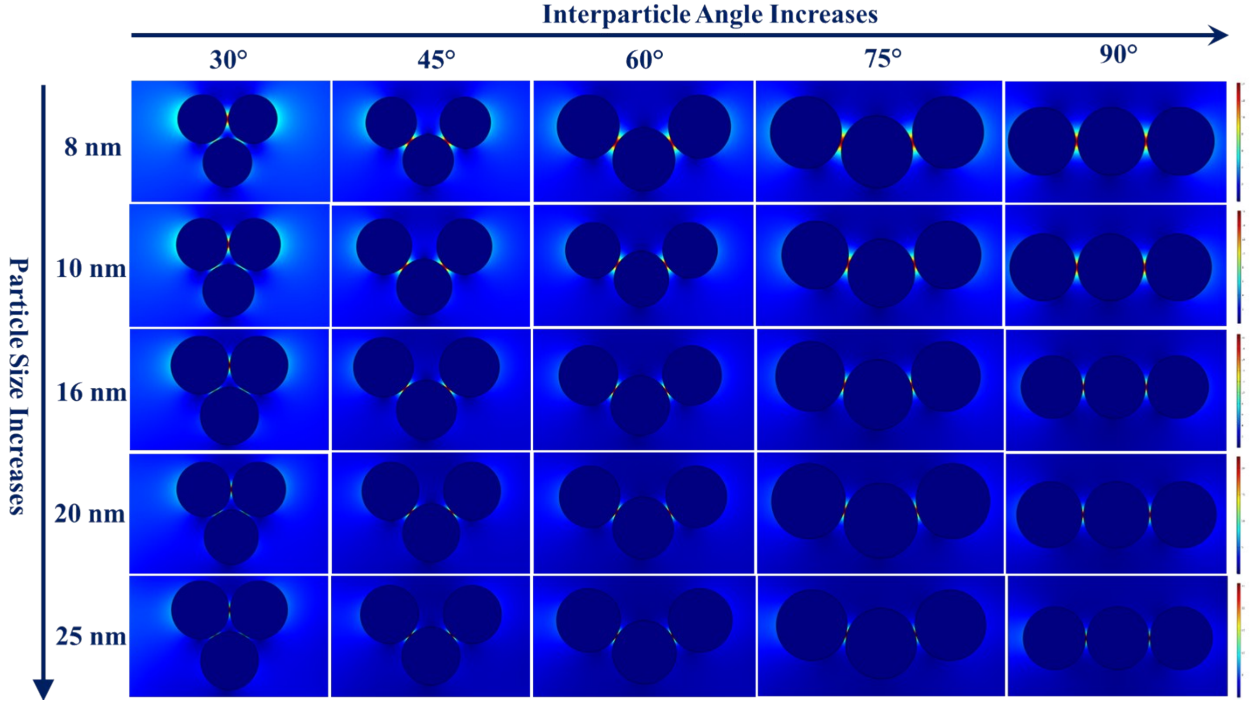

Fig. S5. Electric field distribution pattern for bonding orbitals with increase in interparticle angle of size-specific gold nanoparticles trimers calculated through finite element method.

ESI 7. Enlarged view of the transmission electron micrographs of the gold nanoparticles trimers

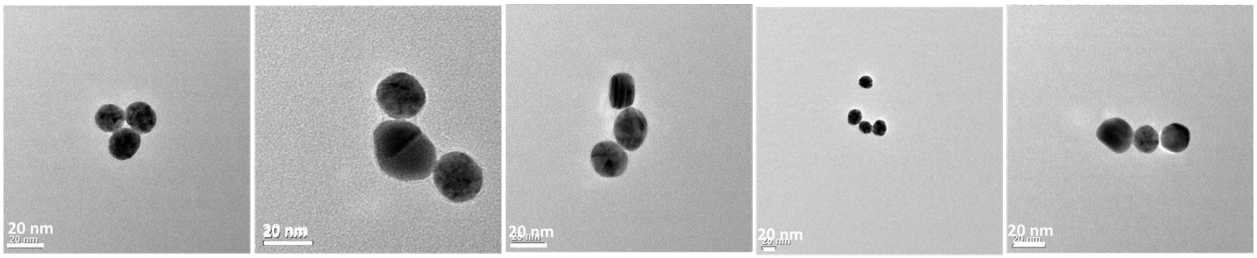

Fig. S6. Enlarged view of the transmission electron micrographs of the gold nanoparticles (set D) trimers
